# Supplementary figures and images for: Mortality Predictors in Severe SARS-CoV-2 Infection
Source: Medicina (Kaunas). 2022 Jul 18;58(7):945. doi: 10.3390/medicina58070945 (PMC9324408; doi:10.3390/medicina58070945)

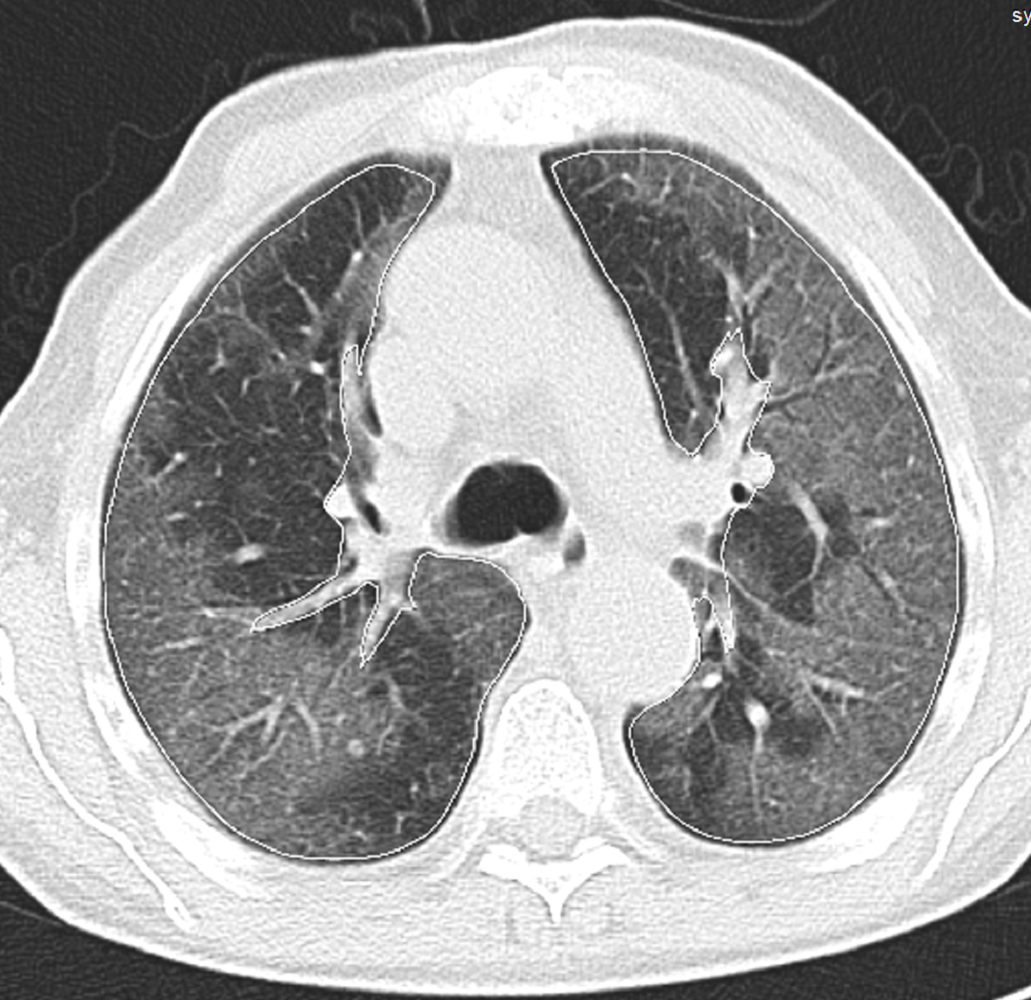

Supplement: Supplementary file 1 [file medicina-58-00945-s001.zip › Figure S1.tif]

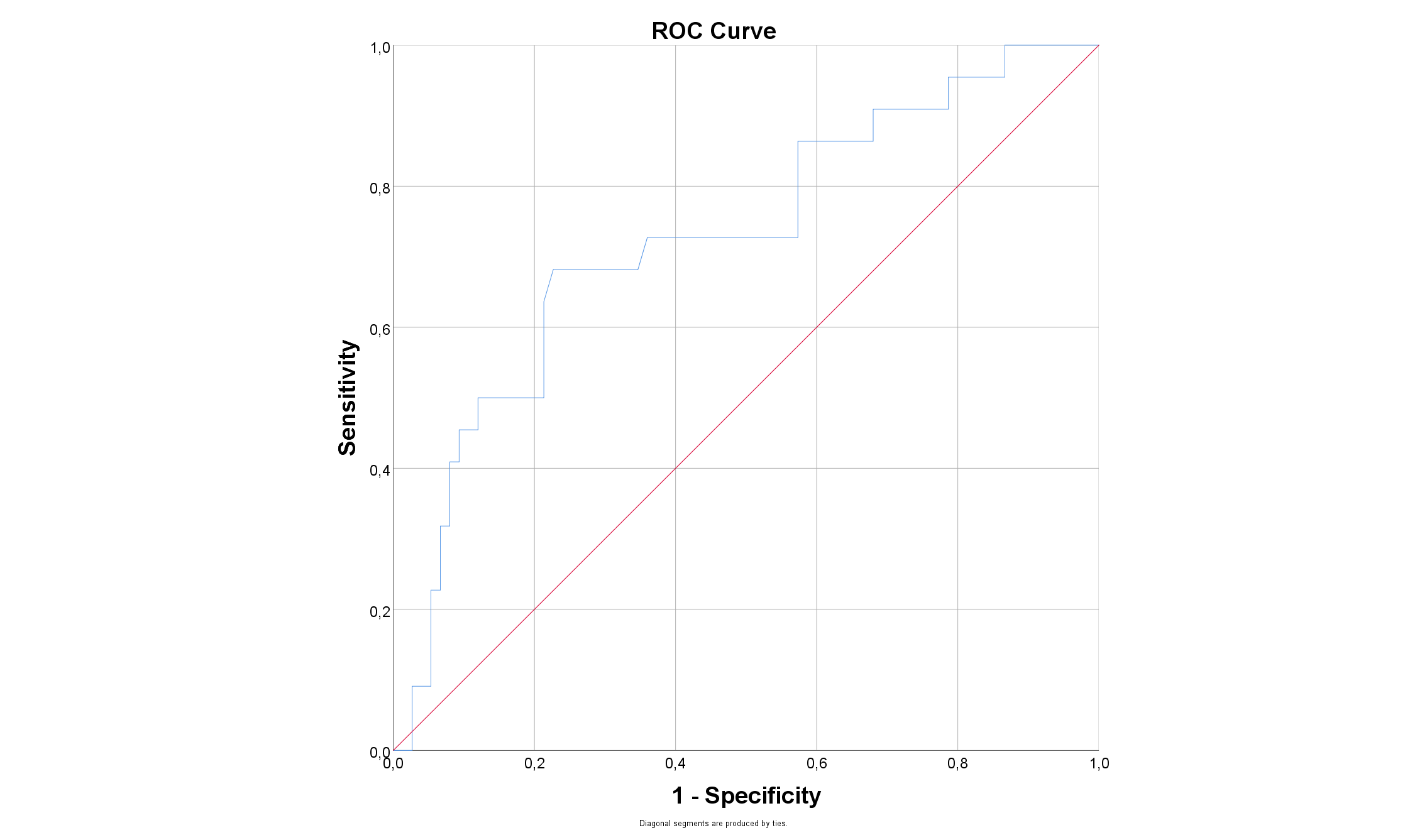

Supplement: Supplementary file 1 [file medicina-58-00945-s001.zip › Figure S10 - D-dimers ROC curve.tif]

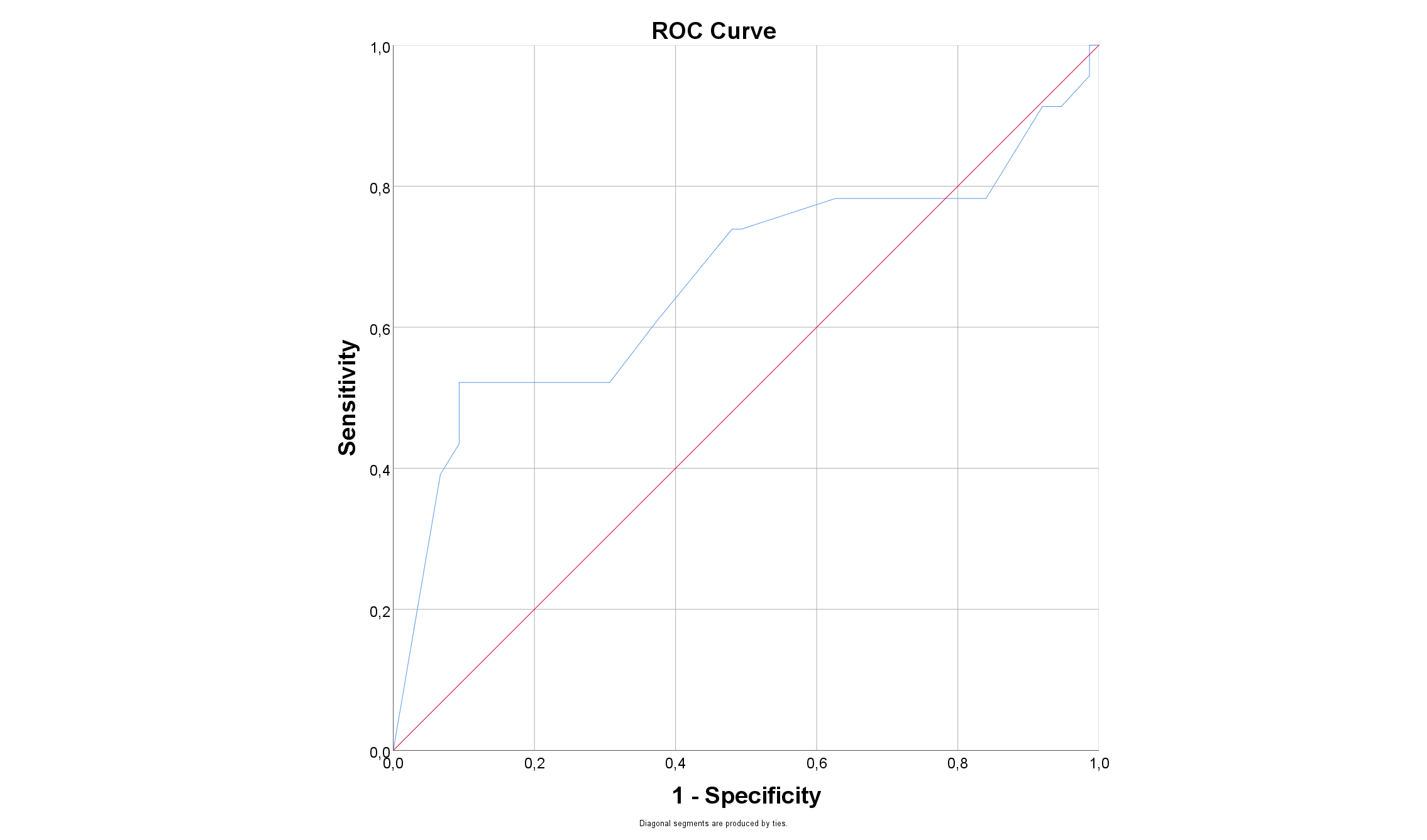

Supplement: Supplementary file 1 [file medicina-58-00945-s001.zip › Figure S11 - Oxygen flow ROC curve.tif]

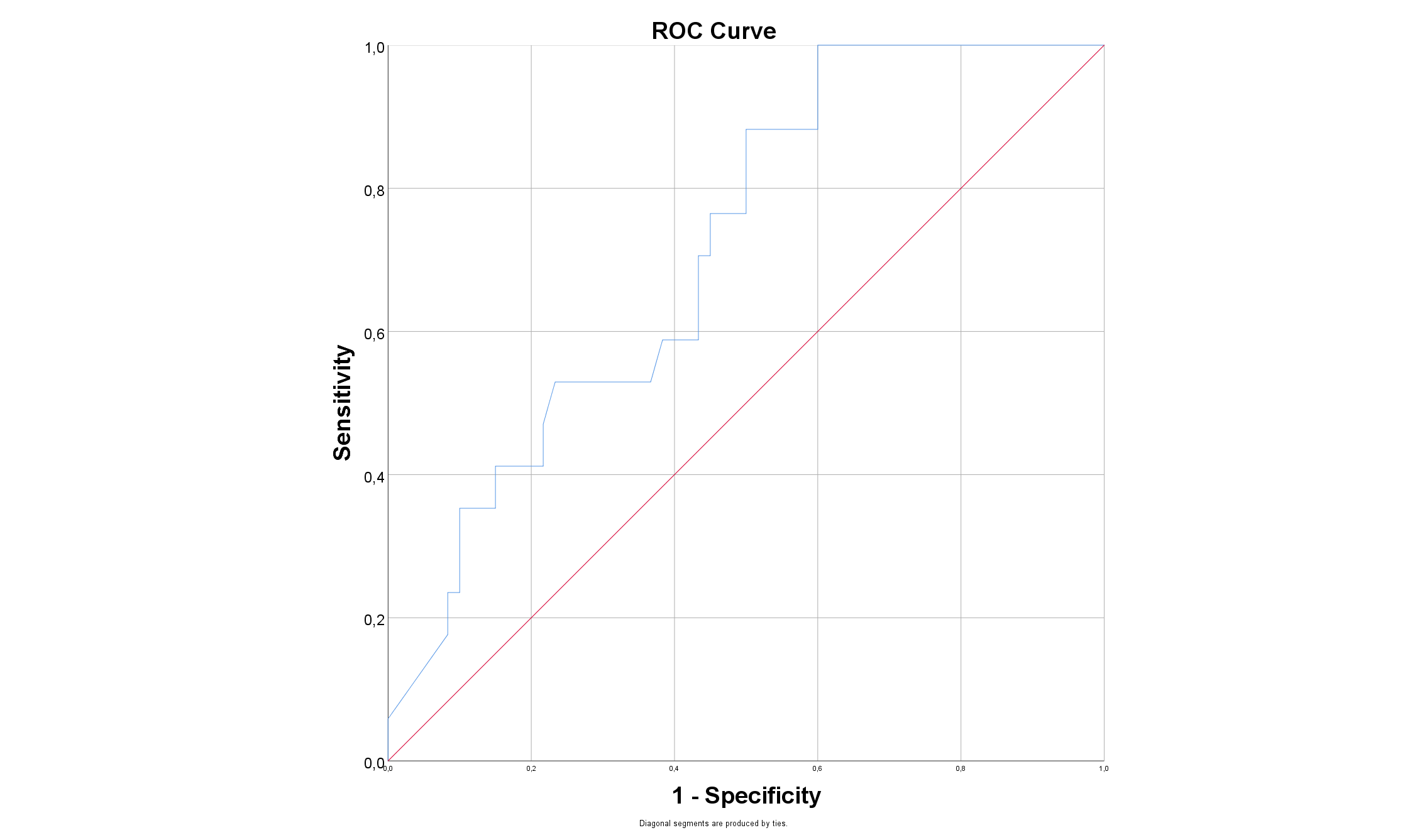

Supplement: Supplementary file 1 [file medicina-58-00945-s001.zip › Figure S12 - Myoglobin ROC curve.tif]

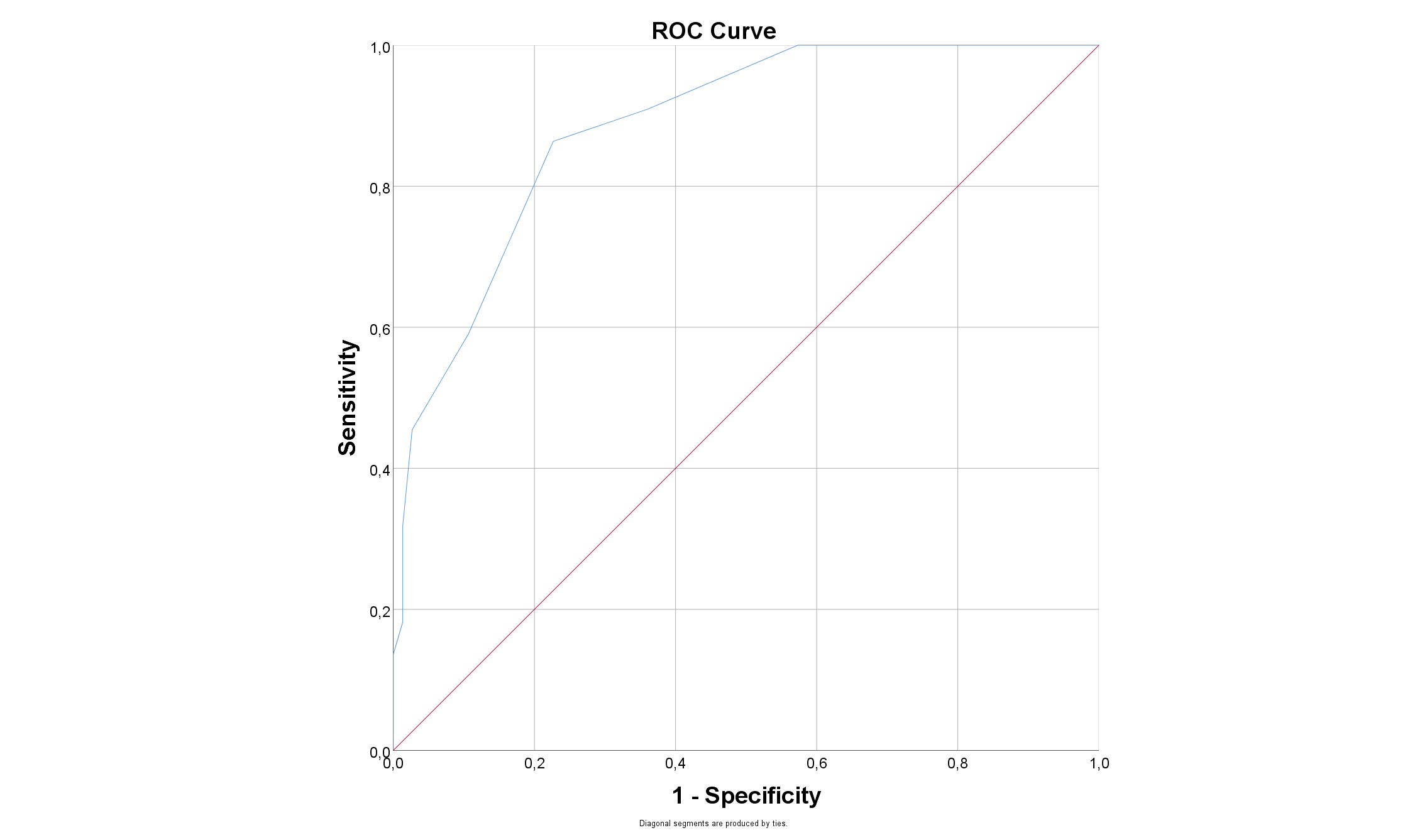

Supplement: Supplementary file 1 [file medicina-58-00945-s001.zip › Figure S13 - COV-Score ROC curve.tif]

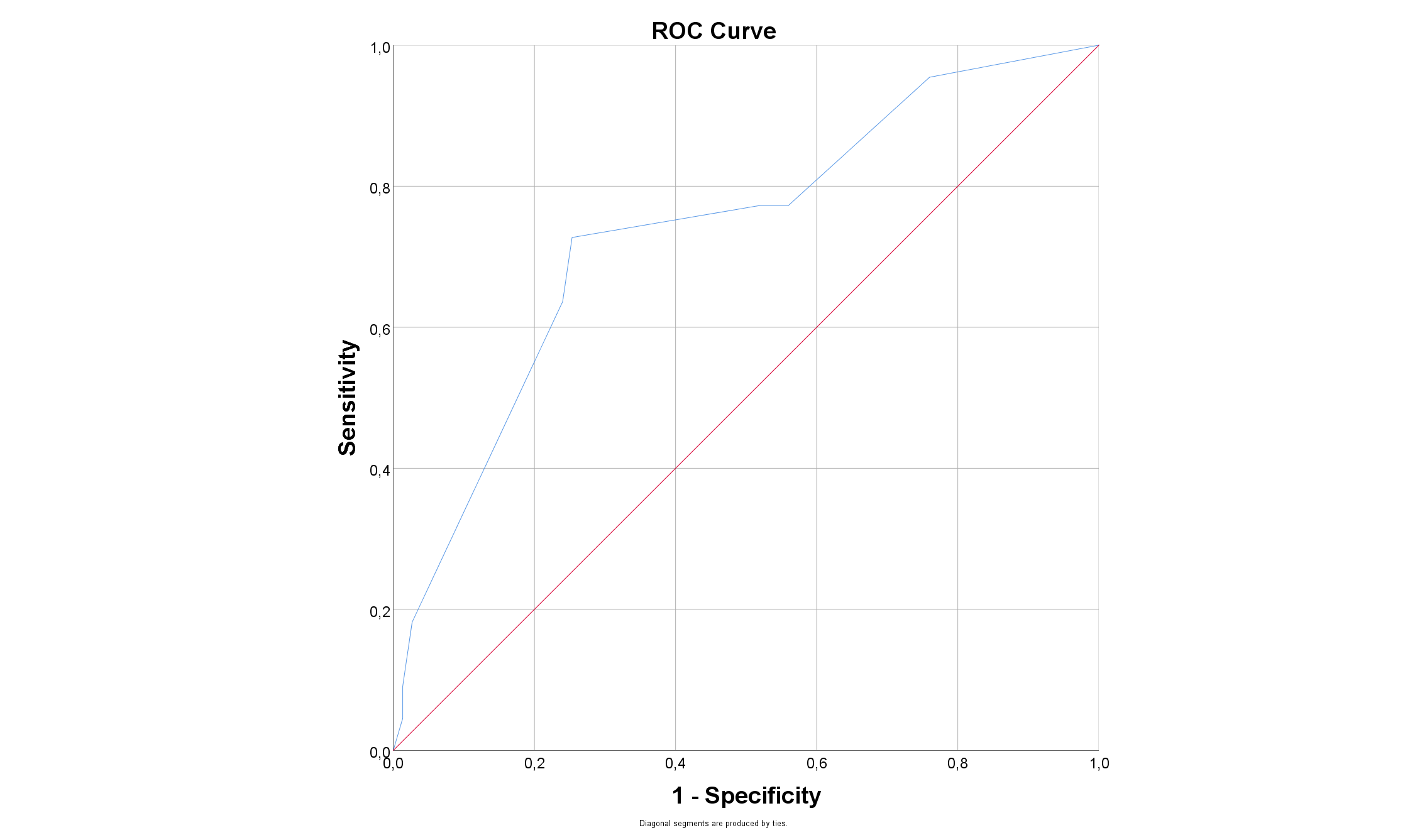

Supplement: Supplementary file 1 [file medicina-58-00945-s001.zip › Figure S14 - MuLBSTA ROC curve.tif]

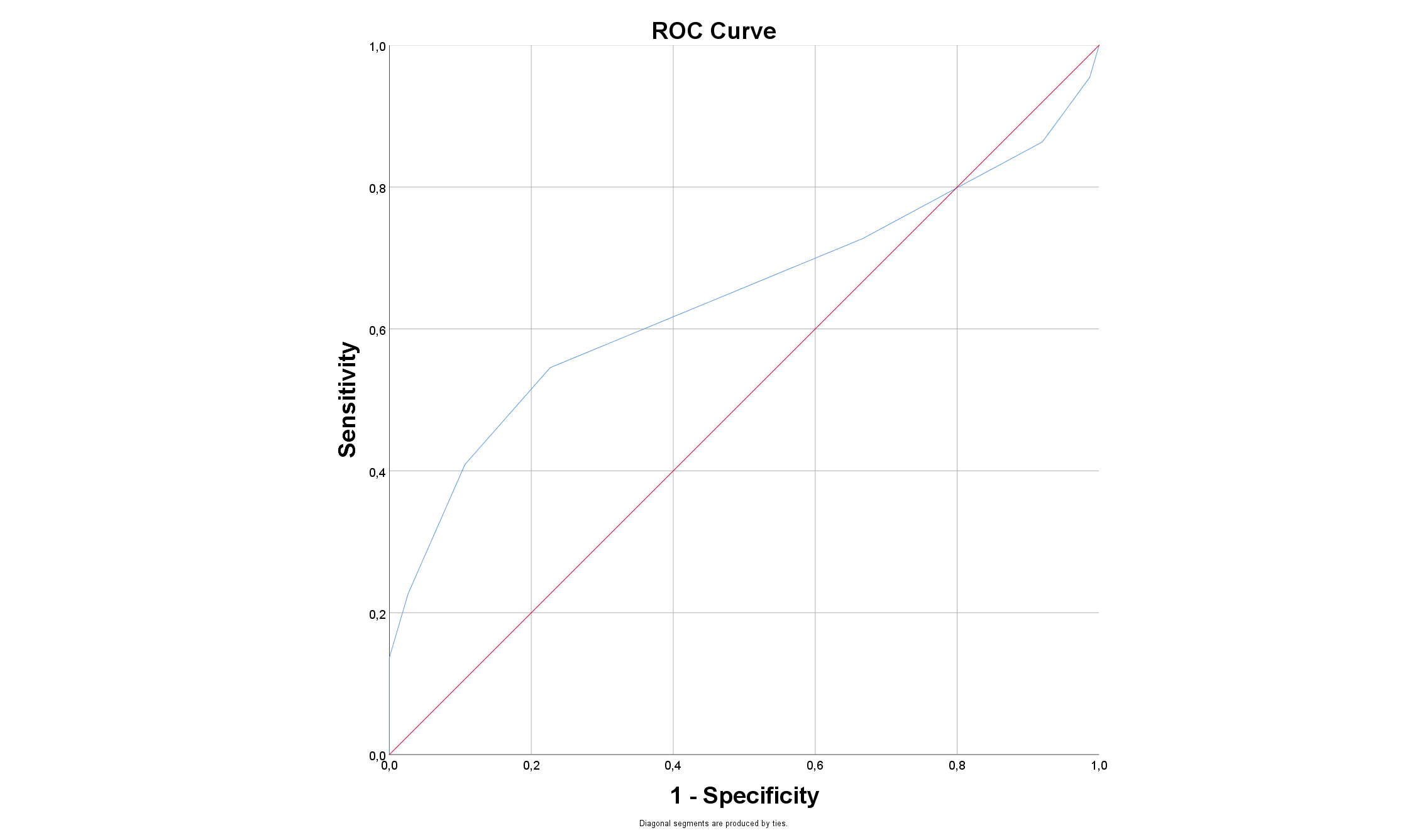

Supplement: Supplementary file 1 [file medicina-58-00945-s001.zip › Figure S15 - Smart COP ROC curve.tif]

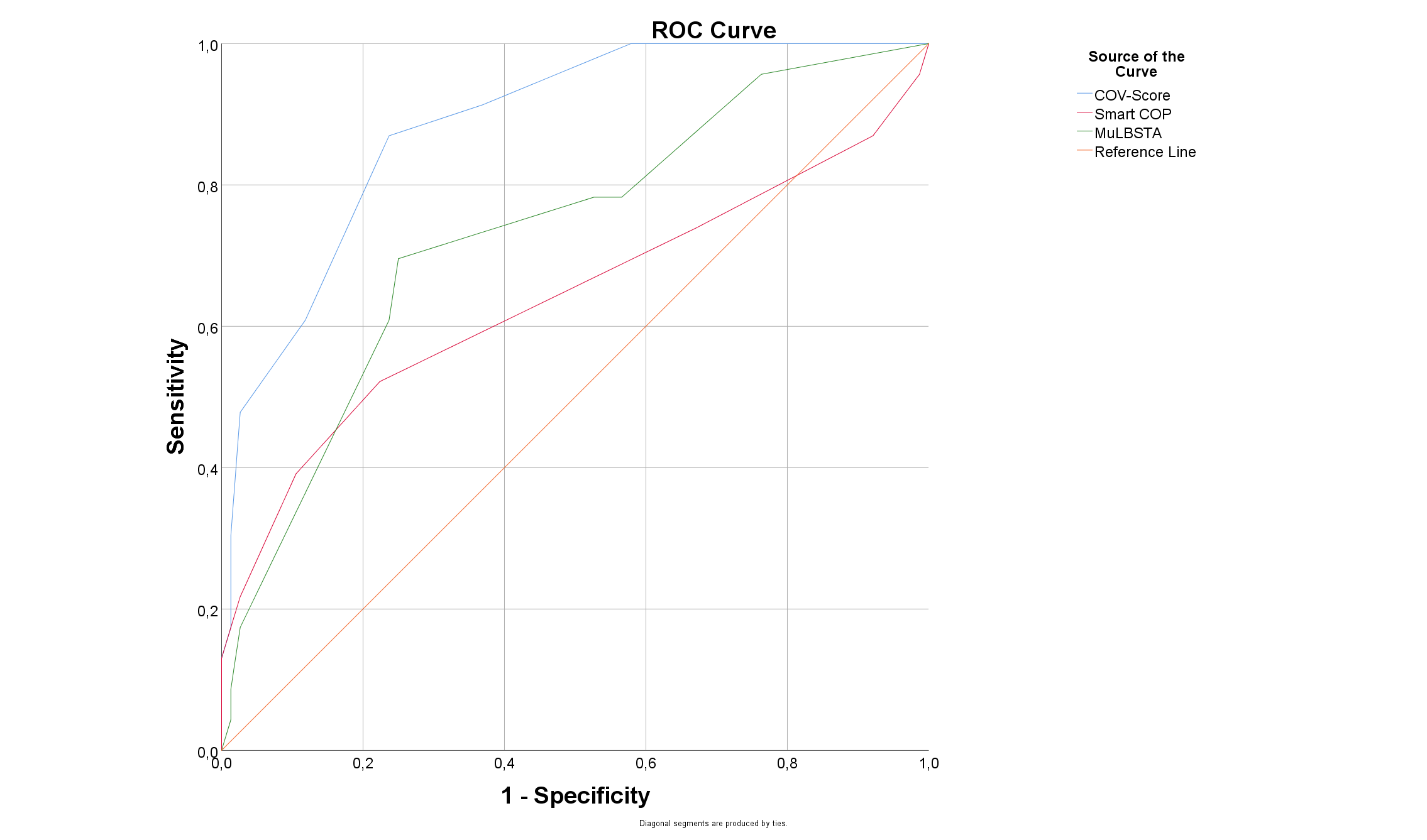

Supplement: Supplementary file 1 [file medicina-58-00945-s001.zip › Figure S16 - ROC curve evaluation for prediction scores.tif]

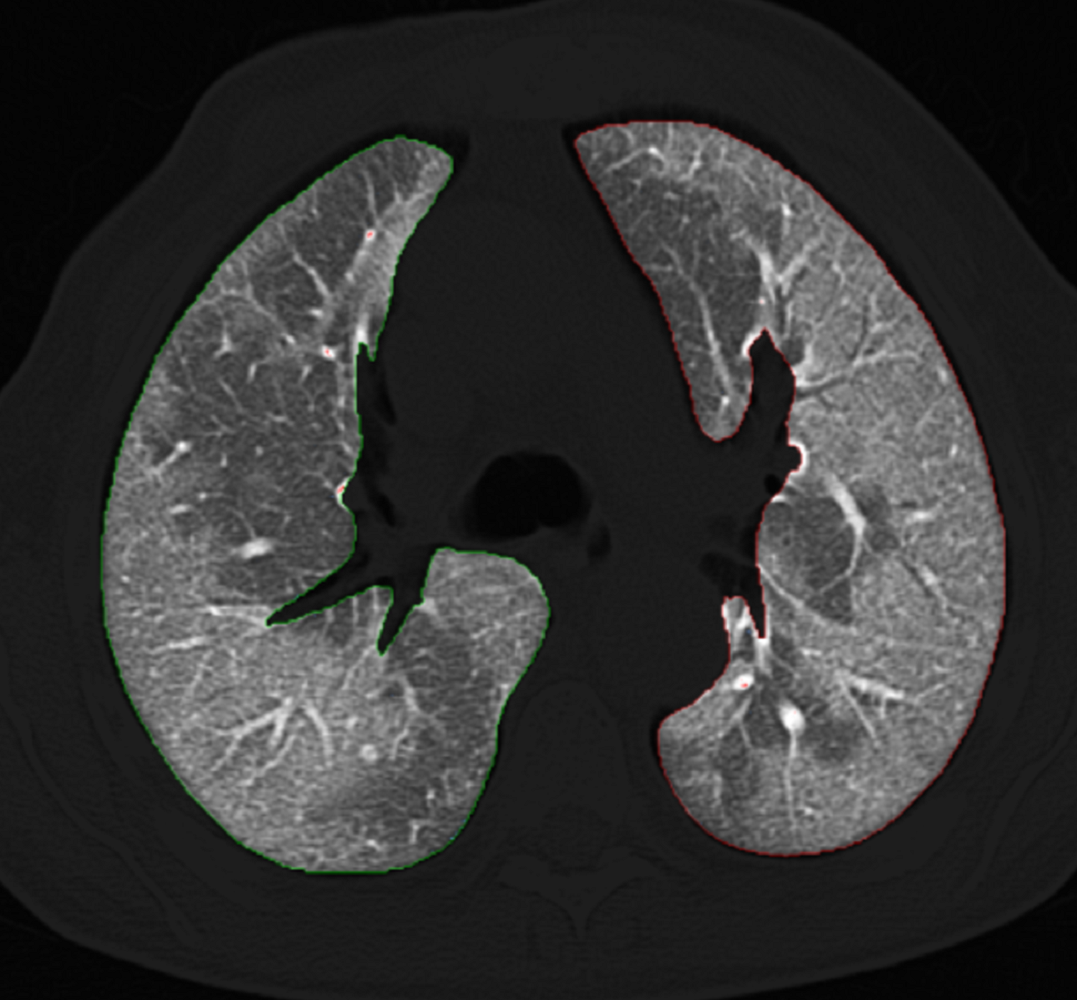

Supplement: Supplementary file 1 [file medicina-58-00945-s001.zip › Figure S2.tif]

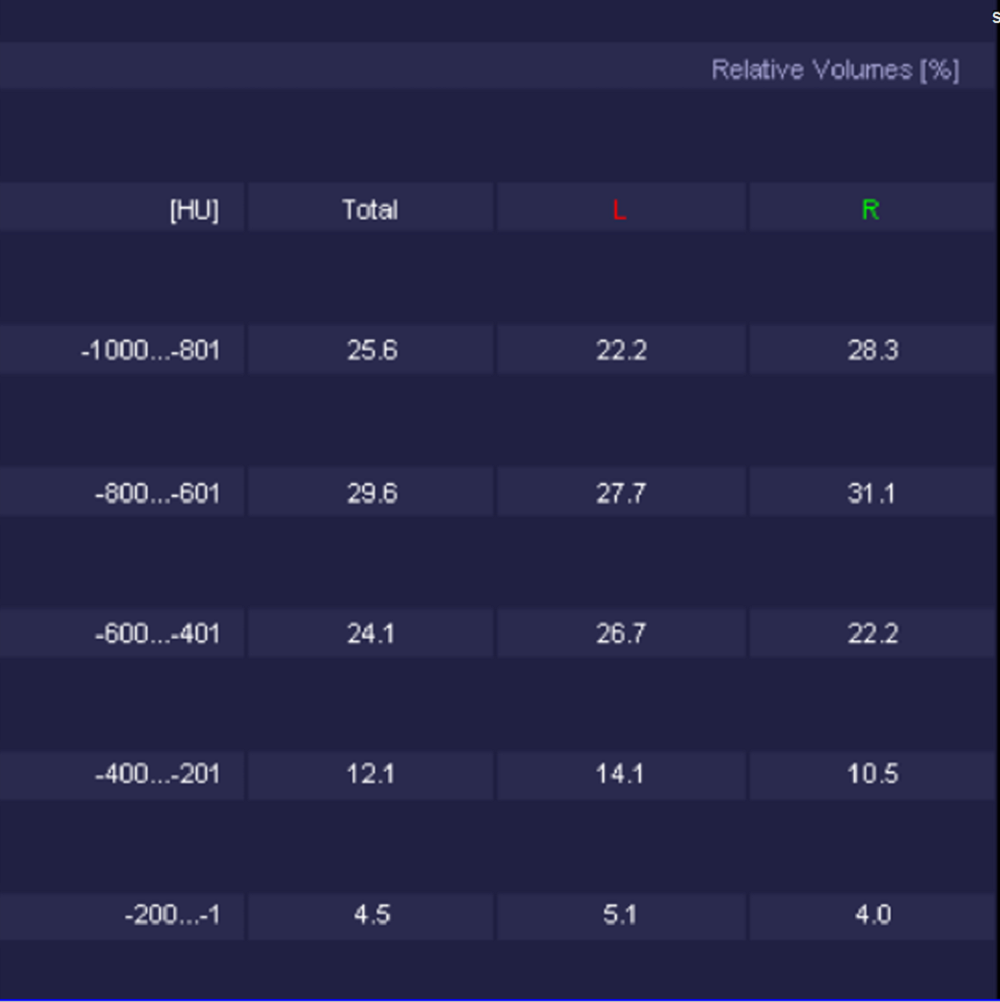

Supplement: Supplementary file 1 [file medicina-58-00945-s001.zip › Figure S3.tif]

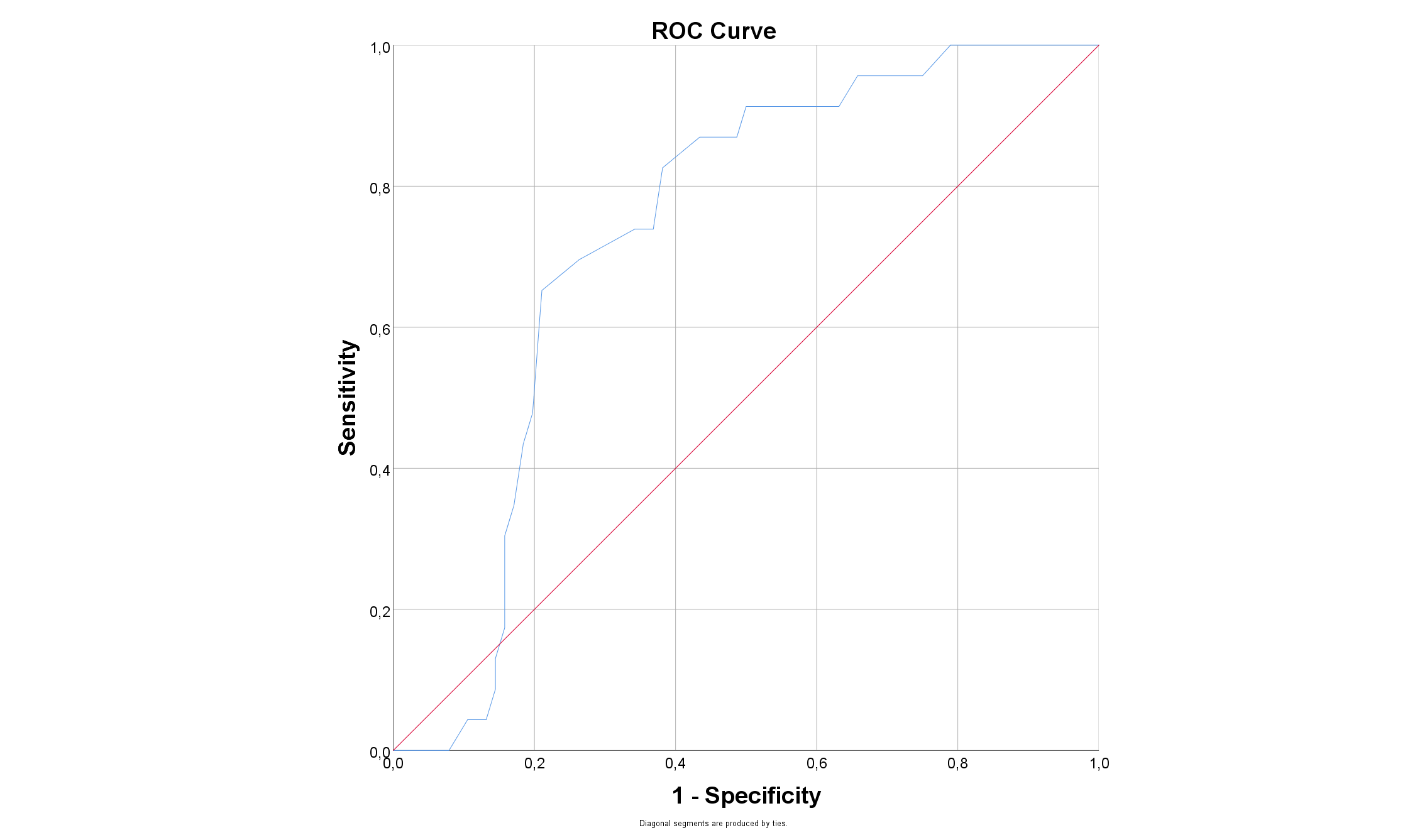

Supplement: Supplementary file 1 [file medicina-58-00945-s001.zip › Figure S4 - Age ROC curve.tif]

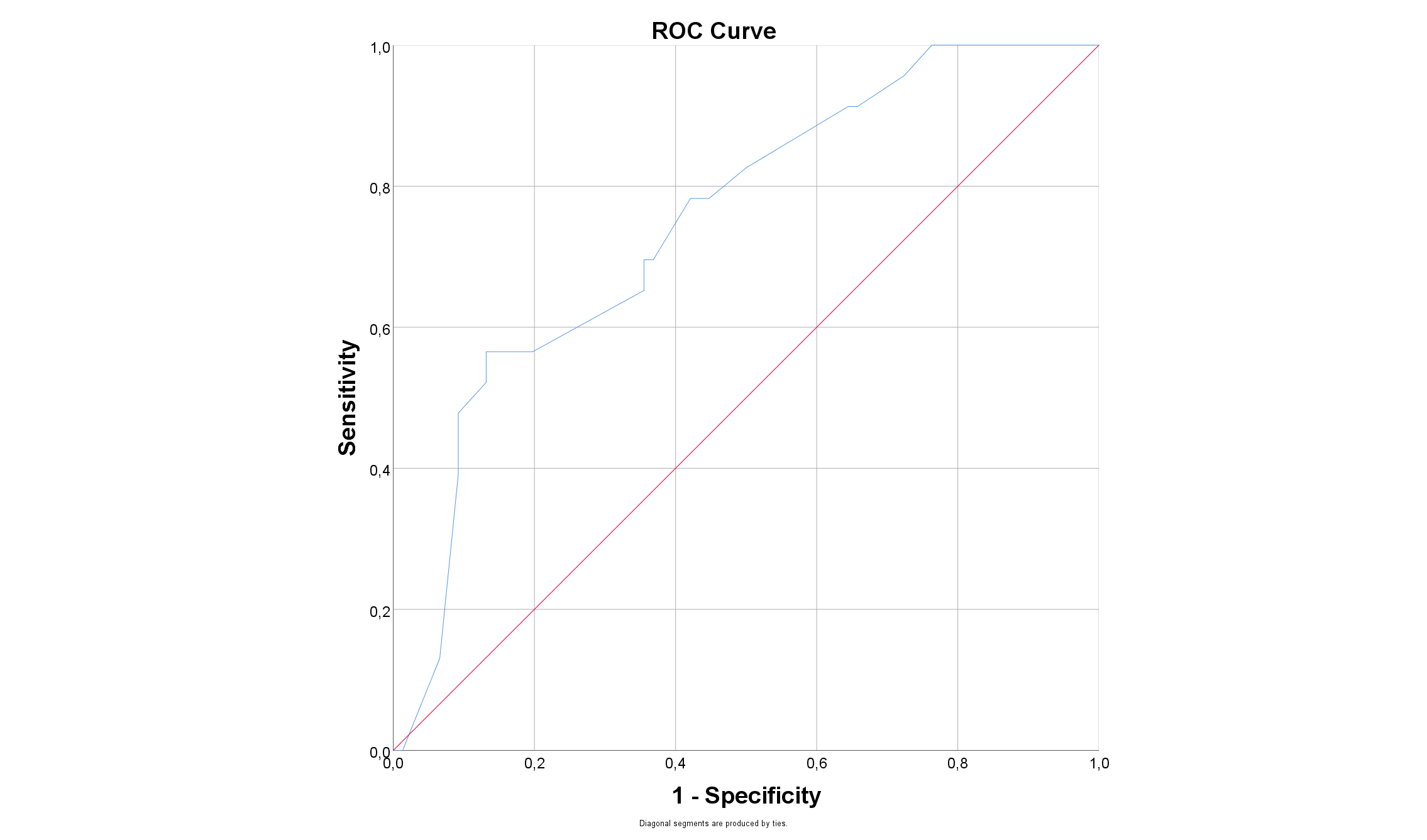

Supplement: Supplementary file 1 [file medicina-58-00945-s001.zip › Figure S5 - Lymphocytes ROC curve.tif]

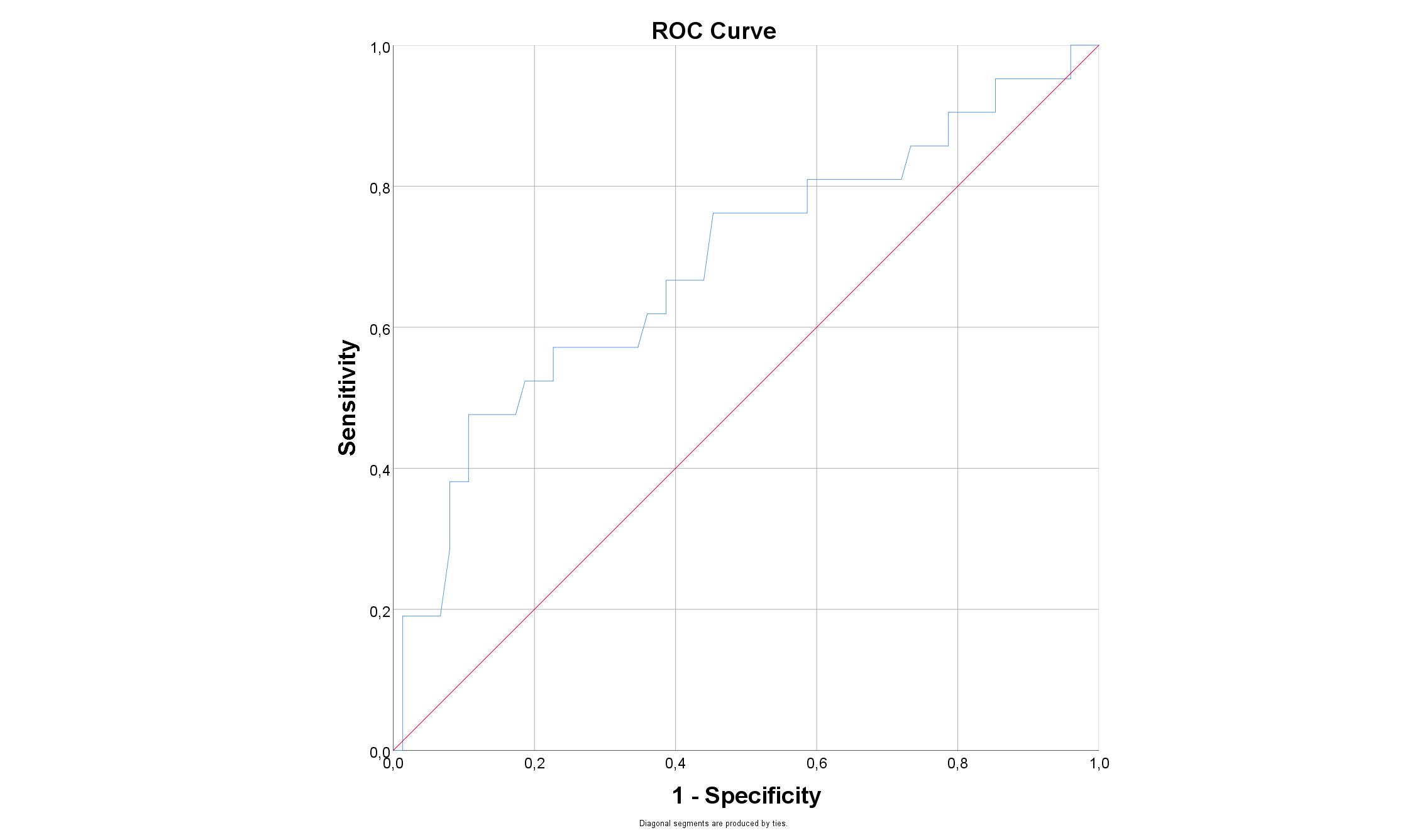

Supplement: Supplementary file 1 [file medicina-58-00945-s001.zip › Figure S6 -PaO2FiO2 ratio ROC curve.tif]

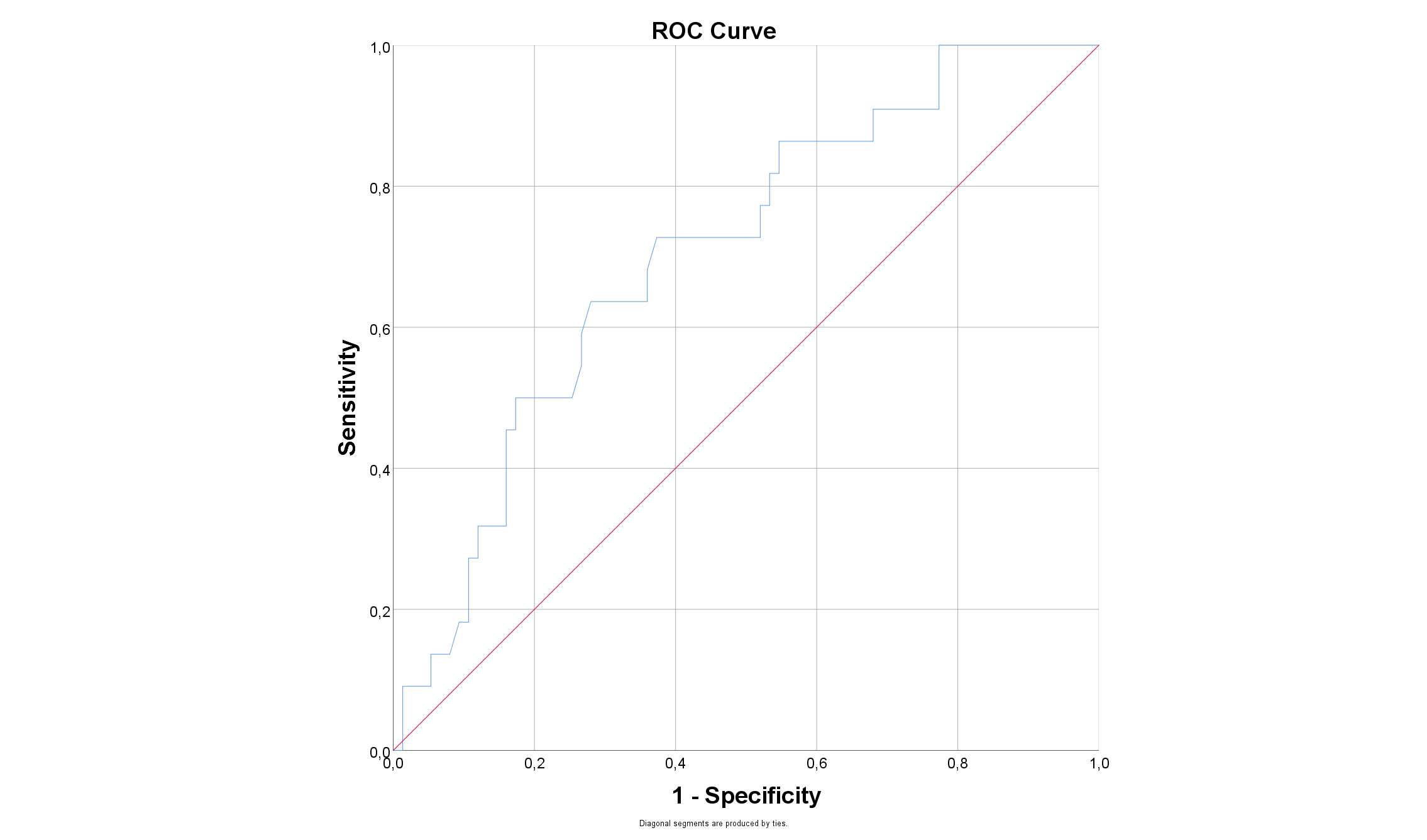

Supplement: Supplementary file 1 [file medicina-58-00945-s001.zip › Figure S7 - Percent of lung involvement ROC curve.tif]

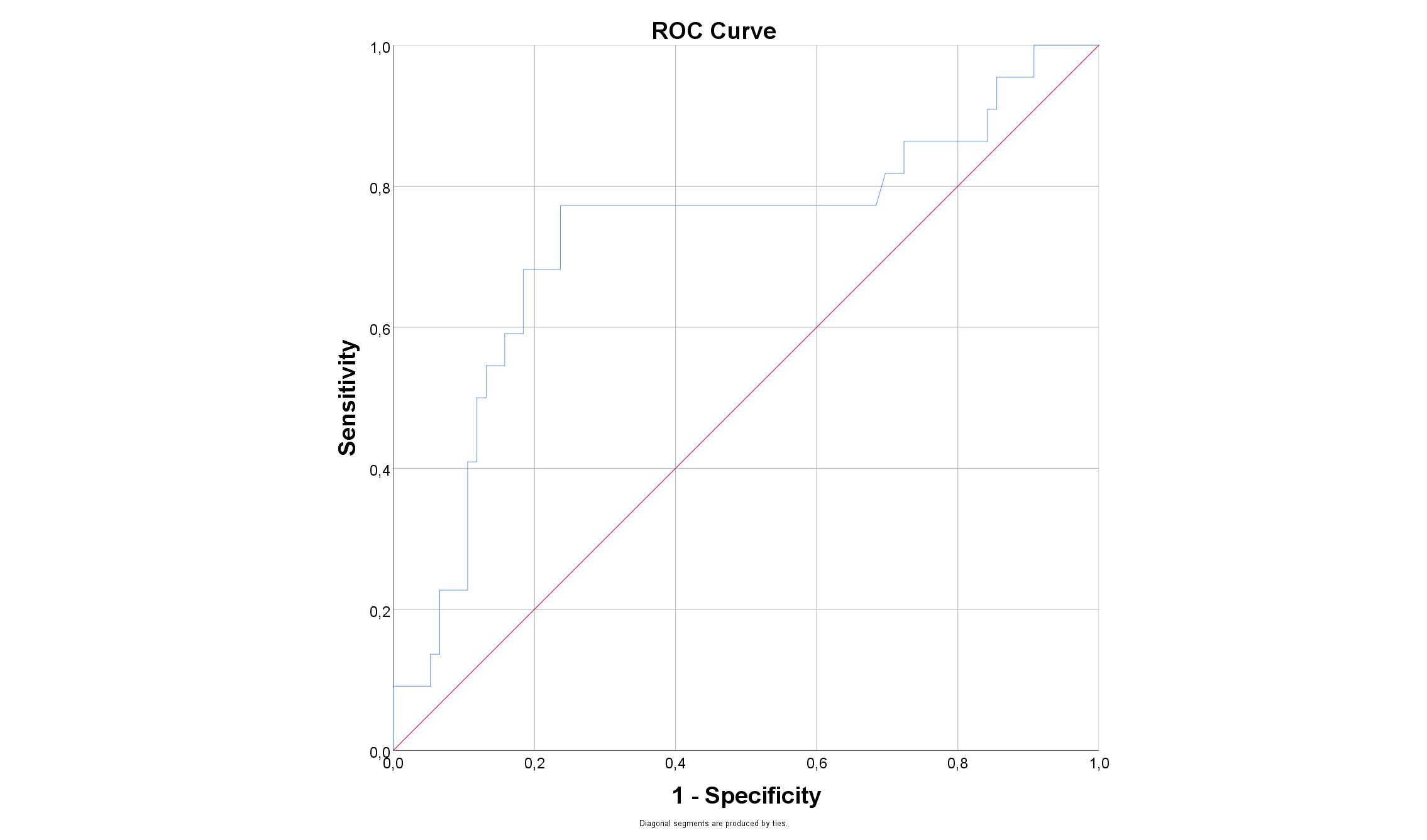

Supplement: Supplementary file 1 [file medicina-58-00945-s001.zip › Figure S8 - Lactate dehydrogenase ROC curve.tif]

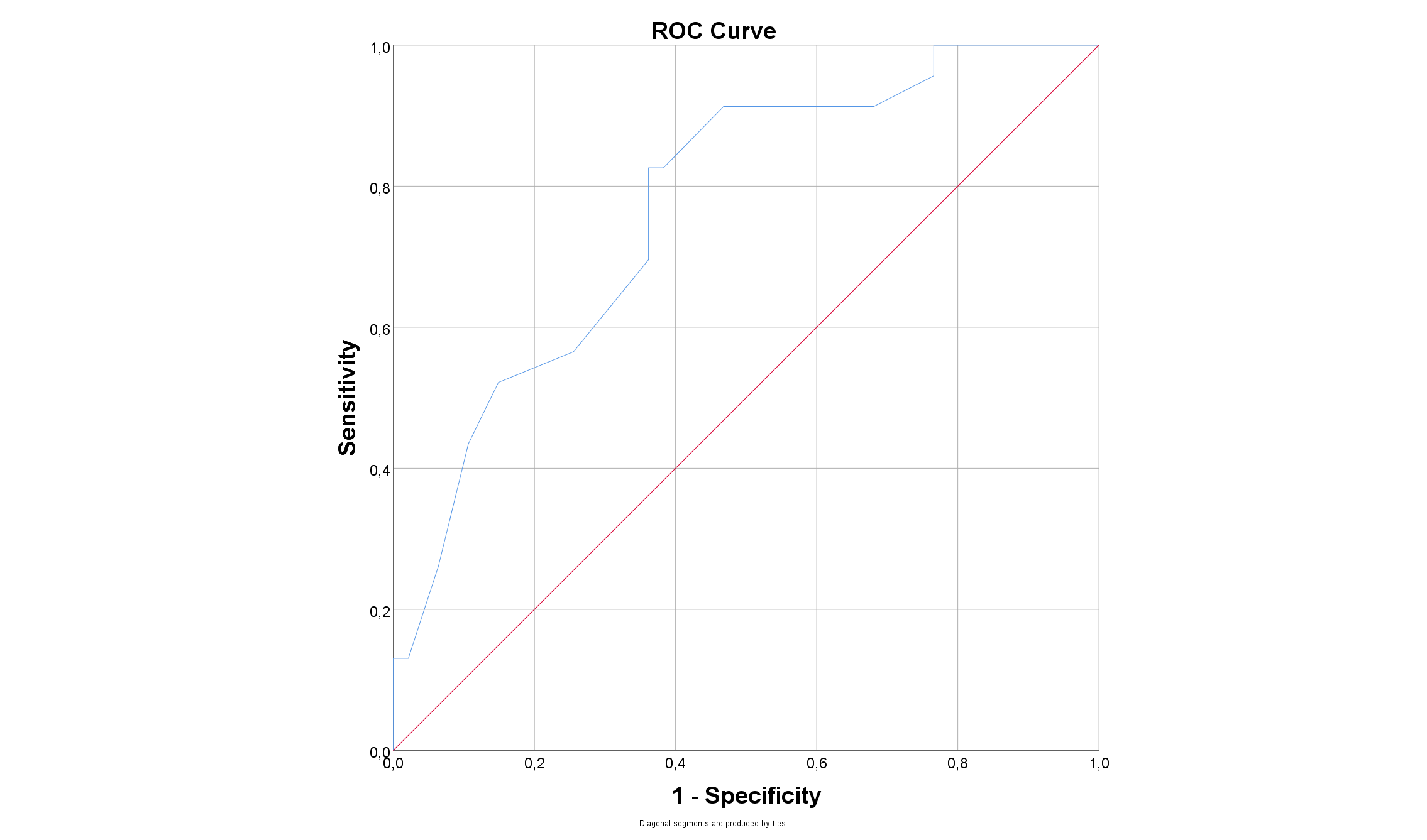

Supplement: Supplementary file 1 [file medicina-58-00945-s001.zip › Figure S9 - Serum albumin ROC curve.tif]
